# miR-365a-3p regulates ADAM10-JAK-STAT signaling to suppress the growth and metastasis of colorectal cancer cells

**DOI:** 10.7150/jca.42731

**Published:** 2020-03-26

**Authors:** Yong-gang Hong, Cheng Xin, Hao Zheng, Zhi-ping Huang, Yuan Yang, Ji-dian Zhou, Xian-hua Gao, Lun Hao, Qi-zhi Liu, Wei Zhang, Li-qiang Hao

**Affiliations:** 1 Department of Colorectal Surgery, Changhai Hospital, Second Military Medical University Shanghai, P.R. China, 200433; 2 Department of Reproductive Heredity Center, Changhai Hospital, Second Military Medical University, Shanghai, 200433, People's Republic of China.; 3 Third Department of Hepatic Surgery, Eastern Hepatobiliary Surgery Hospital, Second Military Medical University, Shanghai, 200438, People's Republic of China.; 4 Key Laboratory of Signalling Regulation and Targeting Therapy of Liver Cancer (SMMU), Ministry of Education, Shanghai, 200438, People's Republic of China.; 5 Shanghai Key Laboratory of Hepatobiliary Tumor Biology (EHBH), Shanghai, 200438, People's Republic of China.; 6 Department of Hepatobiliary Surgery, General Hospital of Southern Theatre Command, Guangzhou 510010, People's Republic of China; 7 Pella Christian High School, Iowa, United States of America.

**Keywords:** miR-365a-3p, colorectal carcinoma, ADAM10, prognosis, biomarker

## Abstract

**Purpose**: MicroRNAs (miRNAs) are key regulators of the growth and development of a wide range of cancer types such as colorectal cancer (CRC). A number of previously studies have observed that the levels of miR-365a-3p expression are dysregulated in many cancers, but the specific role of this miRNA in CRC and its association with patient prognosis remains unclear.

**Methods**: The expression of miR-365a-3p in CRC tissues and cell lines was detected by Real-time Quantitative polymerase chain reaction (RT-qPCR), while the relationship between miR-365a-3p expression and clinicopathological characteristics was further analyzed. After increasing the expression of miR-365a-3p by plasmid transfection in CRC cells, we further investigated the cell proliferation, invasion and migration by cell counting kit-8 (CCK-8), and Transwell assays. Epithelial-mesenchymal transition (EMT) pathway was also measured by western blotting. In addition, the relationship among miR-365a-3p, ADAM10 and JAK in CRC, was explored by luciferase reporter assay.

**Results**: In the present study, we determined that CRC cells and clinical samples exhibited decreased miR-365a-3p expression, and this was associated with larger tumor size, lymph node metastasis, and local invasion. Patients with lower expression of miR-365a-3p had significantly decreased recurrence-free survival (RFS) and overall survival (OS) relative to those with higher levels of this miRNA. In a multivariate analysis, we confirmed that reduced miR-365a-3p levels were independently predictive of poorer CRC patient outcomes. In a functional study, we determined that elevated miR-365a-3p expression inhibited the ability of CRC cells to proliferate and metastasize *in vitro* and *in vivo*. We further identified ADAM10 as a direct miR-365a-3p target, resulting in the suppression of ADAM10 expression in cells expressing this miRNA and ADAM10 levels were in turn closely linked to JAK/STAT signaling.

**Conclusion**: Our study suggested the ability of miR-365a-3p to inhibit the progression of CRC at least in part via suppressing ADAM10 expression and associated JAK/STAT signaling, thus identifying this signaling axis as a possible prognostic and therapeutic target in CRC.

## Introduction

Colorectal carcinoma (CRC) is among the deadliest forms of cancer globally, with particularly pronounced rates of mortality in developed nations 1. Most CRC patients die as a consequence of the progression and metastasis of this tumor type, but the specific mechanisms governing this tumor progression are incompletely understood 2, 3. As such, it is essential that these mechanisms be more fully elucidated in order to facilitate improved care for CRC patients.

MicroRNAs (miRNAs) are short RNA molecules that are unable to encode proteins, but which can still exert biological effects, suppressing or promoting the progression of cancers in a tumor- and miRNA-specific manner via mediating the posttranscriptional suppression and/or degradation of target mRNAs 4. A wide number of miRNAs have recently been shown to serve vital roles in many cancer types including CRC 5, serving as potential diagnostic, prognostic, or therapeutic biomarkers in CRC. Multiple past studies have demonstrated the ability of miR-365 to suppress the progression of cutaneous squamous cell carcinoma 6, lung 7, breast 8, and hepatic carcinoma 9. The exact relevance of this miRNA in CRC, however, remains to be firmly established.

Herein we observed decreased miR-365a-3p expression in CRC patient samples and cell lines. This reduced expression was significantly linked with larger tumors, local invasion and lymph node metastasis, and a poorer overall patient prognosis. When the expression of miR-365a-3p was increased, this impaired the ability of CRC cells to proliferate and metastasize* in vitro* and *in vivo*. We additionally determined that miR-365a-3p targets ADAM10 in order to modulate JAK/STAT signaling in CRC cells, thus highlighting this signaling axis as a potential prognostic biomarker of CRC patient outcomes.

## Materials and Methods

### Patient samples

In total, 134 pairs of CRC tumor and matched paracancerous tissue samples were collected between January 2008 and December 2012at the Changhai Hospital, Shanghai Second Military Medical University. Patients had not undergone preoperative chemo/radiotherapy, and all samples were snap-frozen prior to storage at -80 °C. All patients provided informed consent, and the Institutional Review Board of the Changhai Hospital, Shanghai Second Military Medical University approved this study. The clinicopathological characteristic of study participants are compiled in **Table 1**.

### Cell culture and transfection

The LS174T, DLD1, HT29, HCT116, SW480, and SW620 CRC cells as well as the control colonic epithelial NCM460 line were obtained from the Cell Bank of Type Culture Collection of Chinese Academy of Sciences (Shanghai, China). Cells were grown in either DMEM or RPMI-1640 supplemented with 10% FBS at 37°C in a 5% CO2 incubator. Cells were passaged every 2-3 days. The miR-365a-3p mimic and matched negative control (NC) constructs were obtained from Gene Pharma (Shanghai, China), and were transfected into HCT116 and SW620 cells with Lipofectamine 2000 (Invitrogen, CA, USA). The entirety of the ADAM10 open reading frame was cloned into the pcDNA3.1(+) vector, which was co-transfected into cells along with miR-365-3p mimic for indicated experiments using Lipofectamine 2000.

### qRT-PCR

TRIzol (TaKaRa, Shiga, Japan) was used to extract total cellular RNA, 1 ug of which was then used with a PrimeScript RT Reagent Kit (TaKaRa) to yield cDNA levels. Stem-loop qRT-PCR was used to assess miRNA expression levels with a TaqMan® miRNA Reverse Transcription Kit (Applied Biosystems, NJ, USA) with a ABI 7500 macine (Applied Biosystems).and U6 used as a normalization control. For mRNA expression analyses, a SYBR Premix Ex Taq kit (TaKaRa) was instead used, with β-actin was a normalization control.

### MTT Assay

Cells were grown in 96-well plates for 0, 12, 24, 36, or 48 h after which 5 mg/ml MTT (Sigma-Aldrich, MO, USA) was added per well and cells were incubated for 4 h. Supernatants were then removed and replaced with 200 uL DMSO, after which OD was measured at 450 nm.

### Colony Formation Assay

A total of 1000 cells per group were added to 10 cm cell culture dished and grown for a 2-week period, after which 1% crystal violet was used to stain cells prior to quantification of colony numbers.

### Immunohistochemistry

Paraffin-embedded tissue samples were stained using antibodies specific for PCNA (ab92552) and Ki67 (ab15580). Next, samples were probed using an appropriate HRP-conjugated secondary antibody, and diaminobenzidine (Dako, CA, USA) was then used for color development, after which samples wer counterstained using hematoxylin (Sigma Chemical Co).

### Migration and Invasion Assays

To assess the migratory and invasive properties of cells, Transwell chambers (Costar, NY, USA) and BioCoat Matrigel invasion chambers (BD Biosciences) were used based on provided directions. A total of 6 random fields were counted per group, with each experiment being repeated in triplicate.

### Murine Xenograft Model

Nude male mice (6 weeks old; n=18) were divided into two equal groups at random, and then received an injection of 4×10^5^ HCT116^miR-365a-3p^ or HCT116^NC^ cells in the tail vein in order to model pulmonary metastasis. After 10 weeks, these animals were then euthanized and the number of metastatic lesions in the lungs were counted via microscope following H&E staining. All animals were housed under standard conditions consistent with the guidelines of the Second Military Medical University Animal Care Facility and the National Institutes of Health. For subcutaneous models, mice instead received bilateral subcutaneous implants with 5×10^6^ HCT116^miR-365a-3p^ or HCT116^NC^ cells, with tumor growth being monitored twice per week using calipers. Tumor volume was determined as follows: larger diameter smaller× (diameter)^2^ / 2. Murine experiments were repeated thrice.

### Western blotting

RIPA (Thermo Fisher) containing 1% protease inhibitors (Sigma) was used to lyse cells, which were then spun for 20 min at 16,000 × g at 4 °C. Supernatant protein levels were quantified via BCA assay, and protein aliquots were denatured for 10 minutes in loading buffer prior to separation via SDS-PAGE and transfer onto PBDF membranes (Millipore, MA, USA). Blots were blocked using 5% nonfat milk in TBST for 1 h, and were then probed overnight with antibodies specific for ADAM10 (ab124695), STAT3(ab119352), phosphorylation of STAT3 (p-STAT3, ab76315), JAK2 (ab108596), p-JAK2 (ab32101), or β-actin (ab179467) at 4°C, with all antibodies used at a 1:1000 dilution and β-actin serving as a loading control. Blots were washed thrice, then probed for 2 h with an HRP- conjugated secondary antibody (#7074, 1:10000, Cell Signaling Technology). Enhanced chemiluminescence (Thermo Fisher) as then used for protein visualization, and data were analyze using Image Lab (Bio-Rad).

### Luciferase activity assay

WT or mutated forms of the ADAM10 3'-UTR were cloned into the pGL3 Luciferase reporter vector. HCT116/SW620 cells were then co-transfected with a Renilla plasmid and either of these pGL3-ADAM10-3'-UTR vectors, along with miR-365a-3p mimics or controls using Lipofectamine 2000 Following a 24 h incubation, a Luciferase assay system (Promega, WI, USA) was used based on provided directions.

### Statistical analysis

Data are means ± standard deviation (SD). GraphPad Prism 5.0 (USA) was used for statistical testing. Experiments were repeated thrice. Numerical data were compared via Student's t-tests and one-way ANOVAs as appropriate, while the association between miRNA levels and clinicopathological parameters was assessed via chi-squared test. The Kaplan-Meier approach and log-rank tests were used for comparing survival data. Cox multivariate proportional hazard regression models were conducted in a stepwise forward likelihood ration fashion. P < 0.05 was the significance threshold.

## Results

### CRC samples exhibit miR-365a-3p downregulation

**Figure 1:**
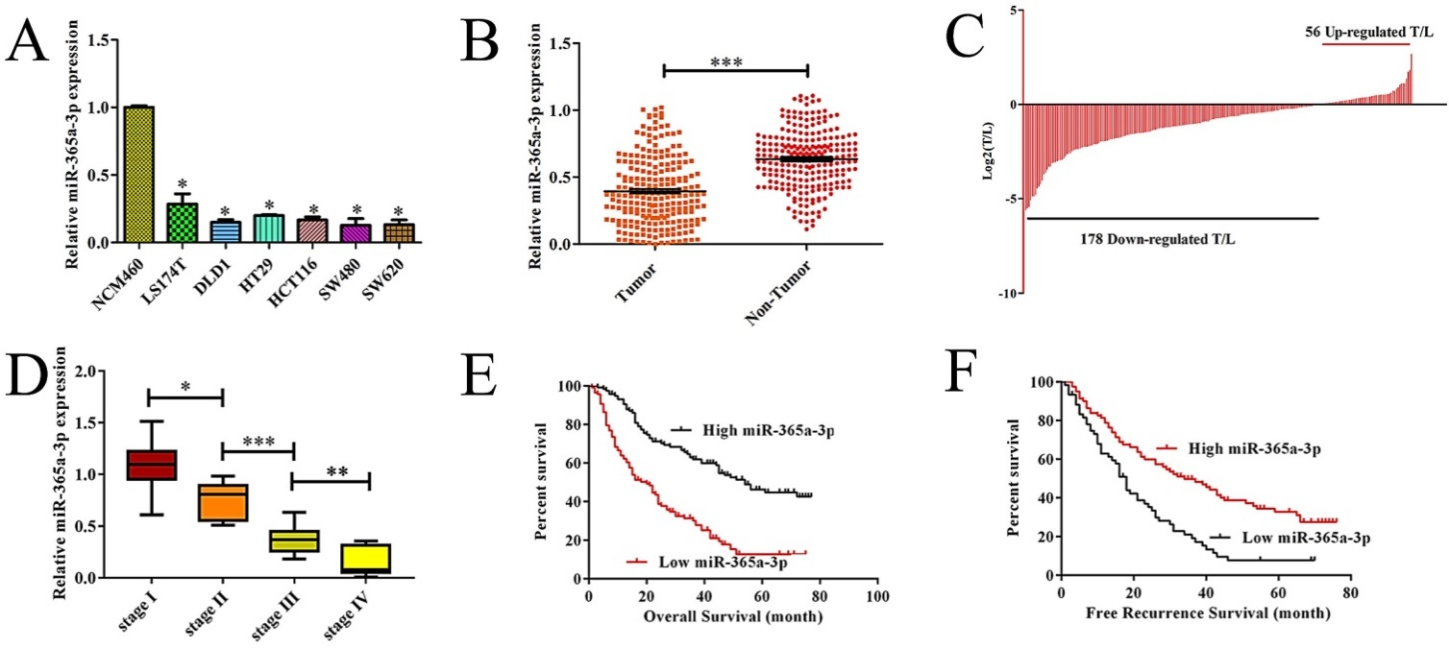
** Reduced expression of miR-365a-3p is associated with more aggressive CRC and a poorer patient prognosis.** (**A**)Relative miR-365a-3p expression CRC cell lines and NCM460 cells as accessed via qRT-PCR. (**B**-**C**) Relative miR-365a-3p levels in 134 pairs of primary CRC patient tumor and paracancerous tissue as accessed via qRT-PCR. (T, tumor; ANT, adjacent nontumor tissue). (**D**) A gradual reduction in levels of miR-365a-3p was evident with increasing TNM stage (I-IV). (**E and F**) The association between levels of miR-365a-3p and recurrence-free survival (RFS,** E**) and overall survival (OS,** F**) in these 134 patients was measured via the Kaplan-Meier method.*p < 0.05; **p < 0.01; ***p < 0.001.

To explore the relevance of miR-365-3p to CRC, we first assessed the expression of this miRNA in a range of different CRC cell lines as compared to control NCM460 colonic epithelial cells. We found that there was a significant decrease in the expression of miR-365-3p in CRC cells relative to these control cells (**Fig. 1A, P<0.05 vs. NCM460**). We further found that when we compared 134 pairs of CRC and control samples from humans, there were markedly lower levels of miR-365-3p in the tumor samples than in the matched paracancerous tissues (**Fig. 1B**), with 79.1% (106/134) of these samples exhibiting reduced expression of this miRNA in tumors relative to matched non-cancerous samples (**Fig. 1C**). We further observed a decrease in the average expression of miR-365-3p as tumor TNM staged rose for I to IV (**Fig. 1D**). Together these findings highlighted the possibility that miR-365-3p may act to suppress CRC development or progression.

### Lower expression of miR-365a-3p is correlated with more aggressive CRC and poorer patient outcomes

We next assessed the relationship between levels of miR-365-3p and the clinicopathologica; characteristics of these 134 CRC patients via separating them into miR-365-3p-high and -low groups based upon the median expression level for this miRNA in this cohort. We found that reduced miR-365-3p levels thus correlated with larger tumor size (P=0.022), local invasion (P=0.043), and lymph node metastasis (P=0.021) (**Table 1**). In contrast, miR-365-3p levels did not significantly correlate with gender (P=0.862), age (P=0.849), tumor location (P=0.073), differentiation grade (P=0.685), TNM stage (P=0.291), serum CA19-9 (P=0.604), or CEA level (P=0.166). Together these findings suggest that reductions in the expression of miR-365-3p may correspond to more aggressive or advanced CRC. We next explore the prognostic relevance of miR-365-3p expression in CRC patients, revealing that patients with low miR-365-3p levels had a markedly shorter recurrence-free survival (RFS, p=0.006,** Fig. 1E**) and overall survival (OS, p=0.026,** Fig. 1F**) that did those patients expressing high levels of this miRNA. In univariate and multivariate analyses, we further confirmed the association between low miR-365-3p expression and decreased RFS and OS in CRC (**Table 2**). Together these findings strongly suggested that miR-365-3p levels were able to independently predict CRC patient prognosis.

### miR-365a-3p overexpression impairs to ability of CRC cells to proliferate *in vitro* and *vivo*

**Figure 2:**
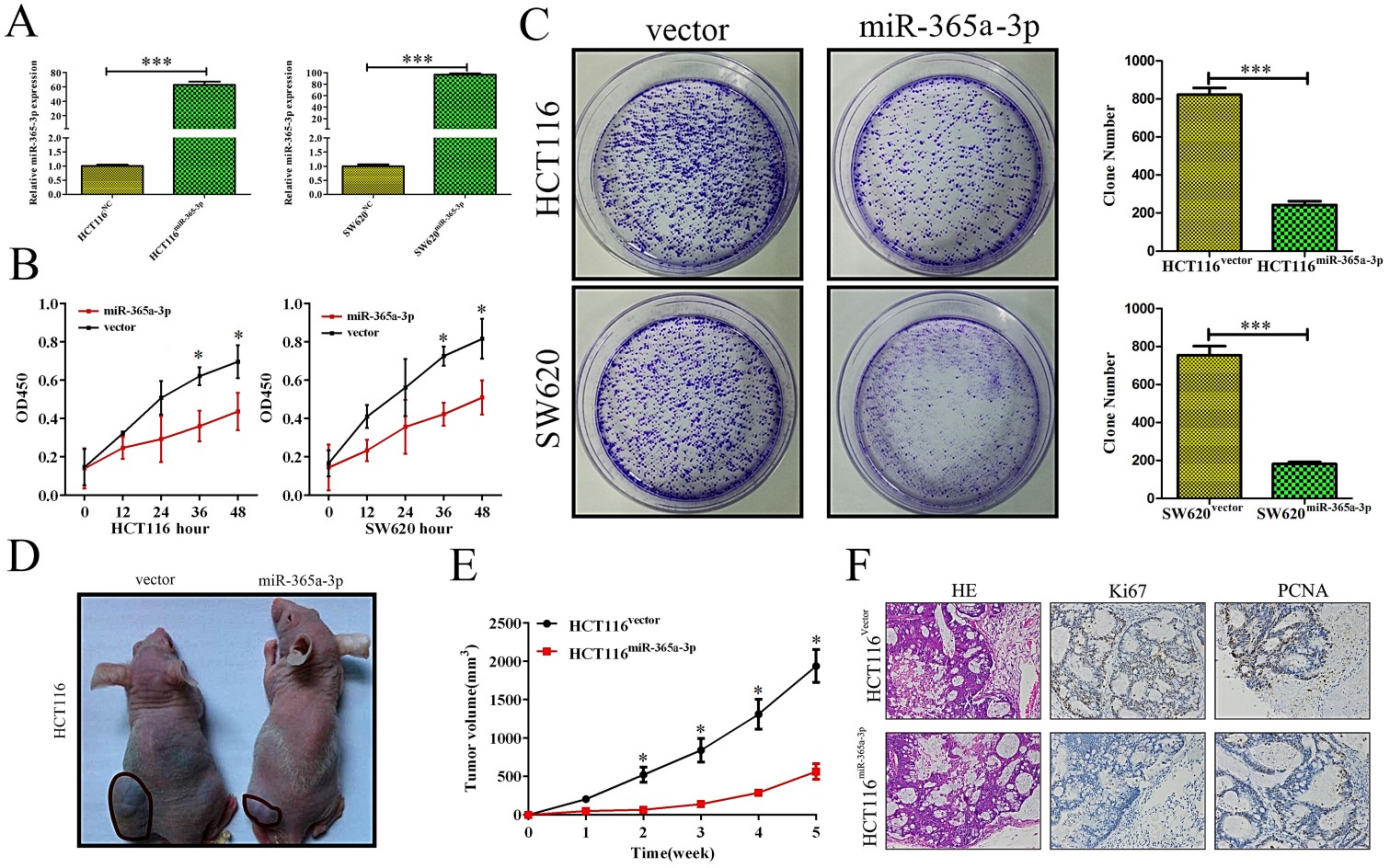
** miR-365a-3p suppresses CRC growth and proliferation.** (**A, B**) HCT116 and SW620 cell viability as accessed via MTT assay. (**C**) A colony formation assay was used to gauge the impact of miR-365a-3p expression on HCT116 and SW620 proliferation. (**D**) Nude mice were subcutaneously implanted with either control or miR-365-3p expressing HCT116 cells, and at the end of tumor growth tumors were excised (n=9). (**E**) Tumor growth curves. (**F**) Tumors were stained for Ki67 and PCNA via immunohistochemistry, with representative images shown (Magnification, ×200). *p < 0.05; **p < 0.01; ***p < 0.001.

As we found reductions in miR-365-3p in CRC to be frequently observed and linked with more aggressive disease, we hypothesized that this miRNA was capable of suppressing the development and/or progression of CRC. We therefore sought to test this hypothesis by transfecting the HCT116 and SW620 CRC cell lines with a miR-365-3p mimic, leading to effective transient upregulation of this miRNA (**Fig. 2A**). We found using MTT and colony formation assays that overexpressing miR-365-3p reduced the ability of CRC cells to grow as compared to cells transfected with a control construct (**Fig. 2B** and **C**). We then sought to confirm this finding *in vivo* by injecting mice with HCT116 cells overexpressing either miR-365-3p or a negative control (NC) vector. We found that the growth of these xenograft tumors was significantly reduced in animals injected using HCT116^miR-365a-3p^ cells as compared with those injected with HCT116^NC^ cells. We additionally isolated xenograft tumors from these animals and stained them for PCNA and Ki67 as markers of proliferation, revealing that the nuclei of HCT116^miR-365a-3p^ xenograft tumor cells were significantly lower for these proliferative markers as compared with those cells from the HCT116^NC^ group (**Fig. 2F**).

### Overexpressing miR-365a-3p impairs to migratory and invasive abilities of CRC cells *in vitro* and* vivo*

**Figure 3:**
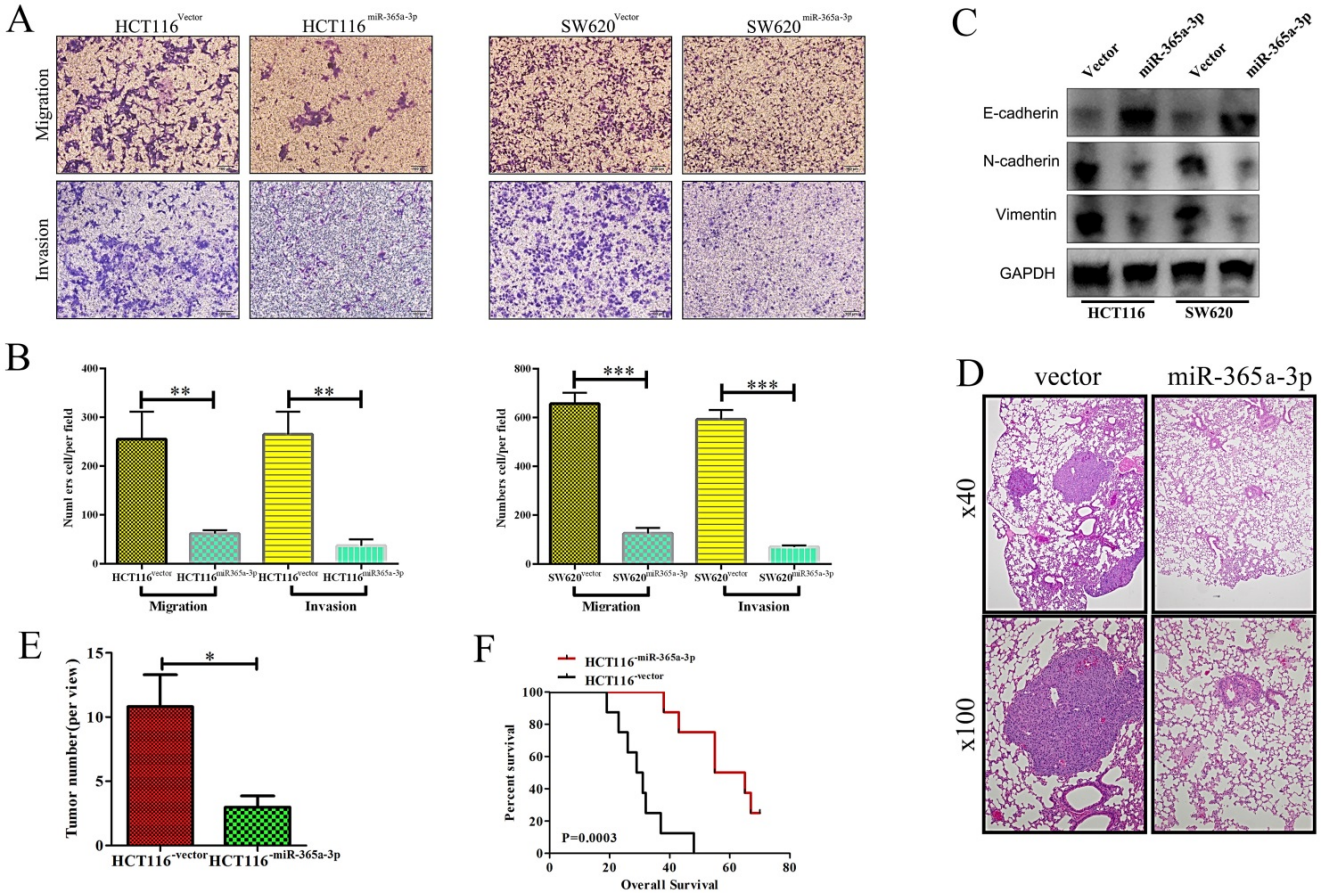
** miR-365a-3p impairs CRC invasion and metastasis.** (**A**) Transwell-based invasion and migration assays were used to assess the impact of miR-365-3p overexpression on HCT116 and SW620 cell migratory and invasive activity, with representative images shown. (**B**) Data from A, quantified as means ± SEM from triplicate experiments. (**C**) Levels of E-cadherin, N-cadherin, and vimentin were measured via Western blotting in CRC cells overexpressing miR-365a-3p. (**D**) Nude mice were administered a tail vein injection of 4×10^5^ HCT116 cells expressing either NC or miR-365-3p (n=9 mice), and after 10 weeks the number of lung metastases in each animal was assessed via microscopic examination, with representative H&E stained images shown (Upper:×40; Lower: ×100) (**E**) Numbers of lung metastases were quantified as means ± SEM. (**F**) Overall survival for mice in each group was assessed via the Kaplan-Meier approach. *p < 0.05; **p < 0.01; ***p < 0.001.

We next explored how miR-365-3p influenced the migratory and invasive capacity of CRC cells using a transwell assay system. When miR-365-3p was overexpressed, this impaired both CRC cell migration and invasion in both tested cell lines (**Fig. 3A and B**). Furthermore, the expression of E-cadherin was enhanced in miR-365-3p overexpressing cells, whereas the mesenchymal markers vimentin and N-cadherin were downregulated (**Figure 3C**). We additionally explored the impact of miR-365-3p expression in metastatic activity *in vivo* using a murine model of lung metastasis wherein mice received an intravenous administration of either HCT116^NC^ or HCT116^miR-365a-3p^ cells. When mice were evaluated after a 10 week period to enumerate lung metastases, revealing fewer and smaller metastases in those mice injected with HCT116^miR-365a-3p^ cells (**Fig. 3D and E**). The mice administered these miR-365-3p overexpressing cells also survived significantly longer than did mice injected with control HCT116 cells (**Fig. 3F**). Together these results indicated that miR-365-3p was able to suppress CRC invasion, proliferation, and metastasis.

### miR-365a-3p directly targets ADAM10 in CRC

**Figure 4:**
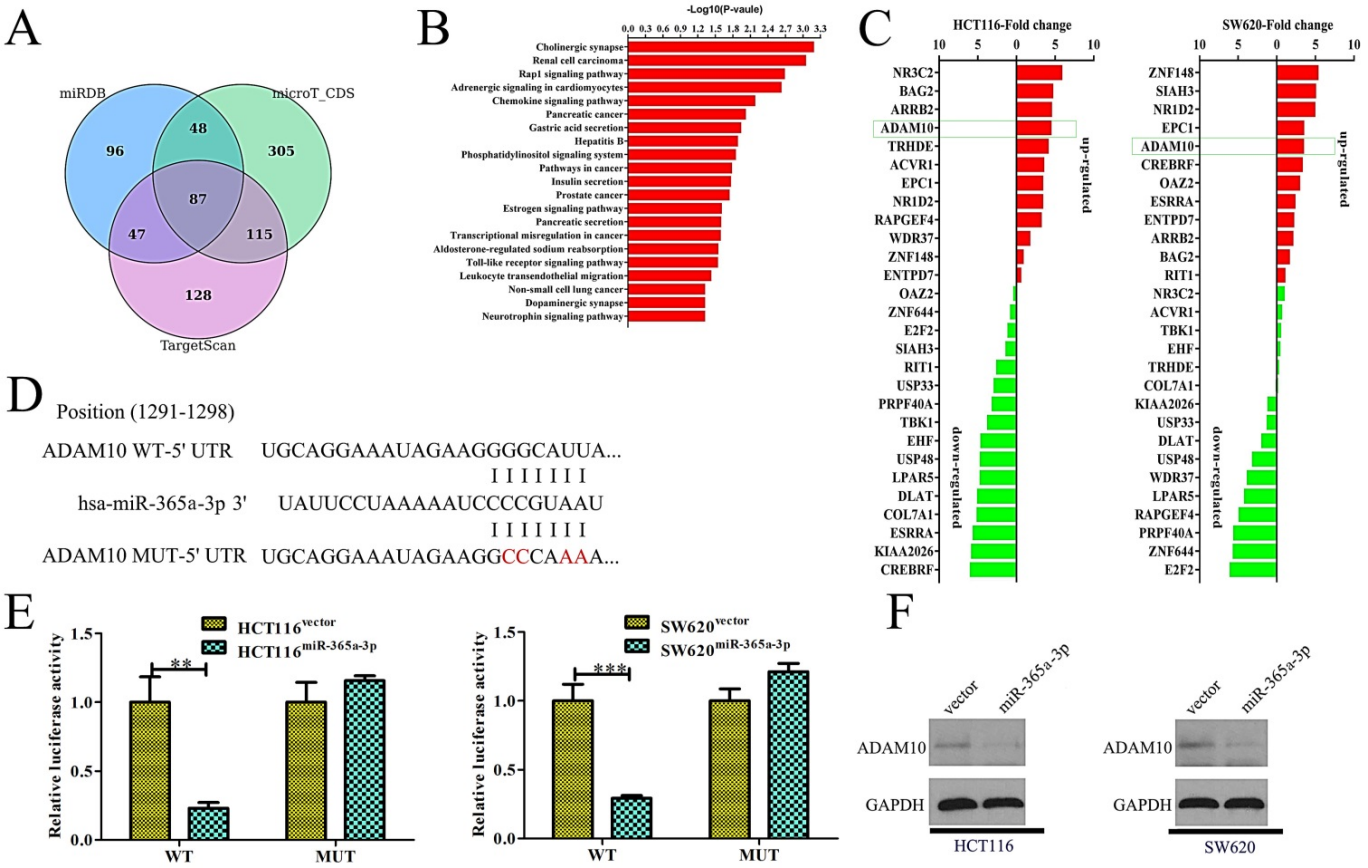
** miR-365a-3p directly targets ADAM10 in CRC.** (**A**)Three bioinformatics tools were used to identify potential miR-365a-3p targets. (**B**) The 81 target genes that overlapped among these predictive tools were validated via qRT-PCR in CRC cells. (**C**) miR-365a-3p and the corresponding predicted binding site in the 3'-UTR of ADAM10, with wild-type (WT) and mutated (Mut) versions of this sequence being shown. (**D**) A clear reduction in luciferase activity was evident in CRC cells overexpressing miR-365-3p and the WT but not the mutated version of the ADAM10 3'-UTR. (**E**) Overexpression of miR-365a-3p in CRC cell lines reduced levels of ADAM10 protein. UTR: untranslated region. *p < 0.05; **p < 0.01; ***p < 0.001.

We next sought to identify targets of miR-365-3p in order to understand how this miRNA influenced CRC proliferation and metastasis. To that end, the microT-CDS, miRDB, and TargetScan bioinformatics tools were used to identify 87 predicted targets shared among these three programs (**Fig. 4A**). Of these 87 genes, we were able to validate significant differences in the expression of just 28 of these genes by qRT-PCR when comparing control and miR-365-3p overexpressing HCT116 and SW620 cells. Of these genes, one of the most significantly suppressed in miR-365-3p overexpressing cells was ADAM10, which is known to be linked with tumor invasion and metastasis 10 (**Fig. 4B**). As such, we next confirmed that miR-365-3p does bind to the 3'-UTR of ADAM10 using a dual-luciferase reporter assay (**Fig. 4C**). We found that miR-365-3p significantly reduced the activity of luciferase plasmids encoding the WT but not the mutated version of this sequence in CRC cells (**Fig. 4D**). Consistent with these findings, miR-365-3p overexpression was associated with reduced ADAM10 protein levels (**Fig. 4E**). These findings thus substantiated ADAM10 as a direct target of miR-365-3p in CRC cells.

### ADAM10 expression reverses the ability of miR-365a-3p to suppress CRC

**Figure 5:**
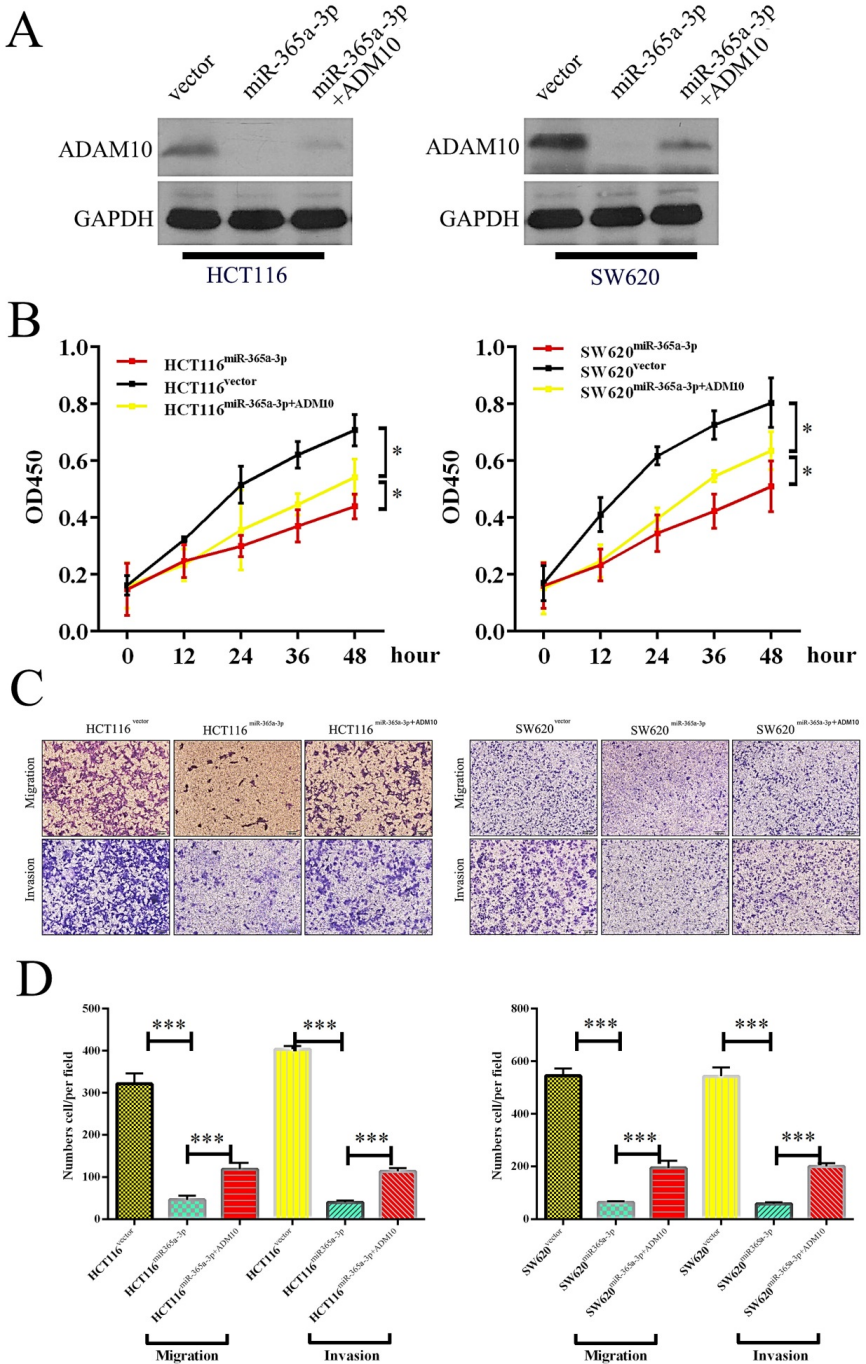
** ADAM10 overexpression enhanced the proliferation and migration of CRC cells, and miR-365a-3p reversed this effect.** (**A**) Levels of ADAM10 protein were assessed in HCT116 cells overexpressing vector control, miR-365p-3p, or miR-365-3p+ADAM10, with GAPDH used as a loading control. (**B**) A CCK8 assay was used to analyze HCT116 cells overexpressing vector control, miR-365p-3p, or miR-365-3p+ADAM10, with data given as means ± SD of 6 replicates per condition. (**C**) HCT116 cell migration was assessed via Transwell assay following the indicated treatment combinations. (**D**) Data from three random fields of view were quantified. *p < 0.05; **p < 0.01; ***p < 0.001.

We next sought to establish whether miR-365-3p was modulating the progression of CRC through its ability to suppress ADAM10 expression by transfecting cells that overexpressed miR-365-3p with a plasmid that mediated either control vector or ADAM10 overexpression (**Fig. 5A**). While miR-365-3p overexpression markedly reduced the proliferation of CRC cells, ADAM10 overexpression reversed this effect and enhanced overall cellular proliferation (**Fig. 5B**). Similarly, ADAM10 overexpression markedly overcame the ability of miR-365-3p to suppress HCT116 and SW620 cell migration and invasion (**Fig. 5C** and **D**).

### miR-365-3p regulates JAK/STAT signaling to suppress CRC progression

**Figure 6:**
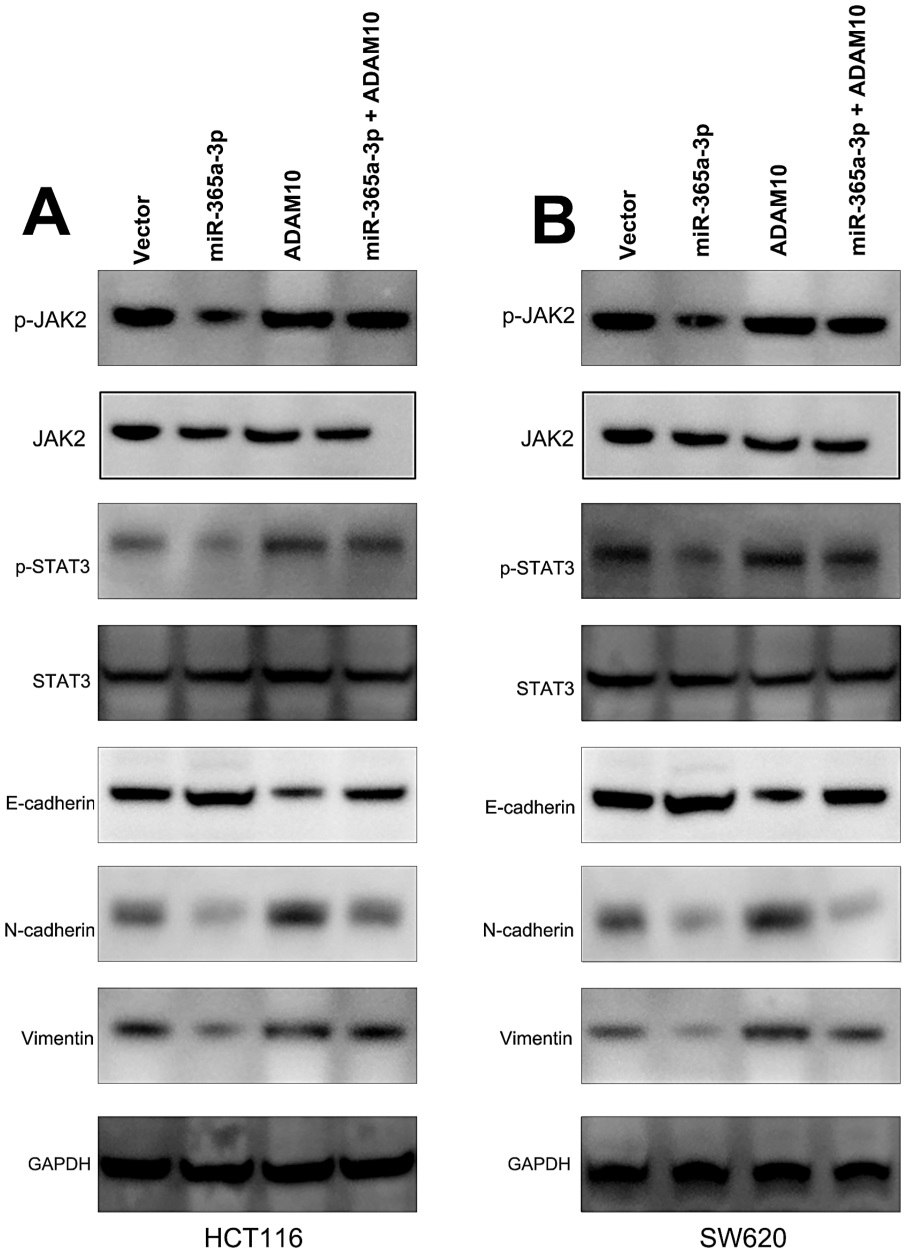
** miR-365-3p regulates JAK/STAT signaling to inhibit CRC progression.** (**A and B**) Western blotting as used to assess p-STAT3, STAT3, p-JAK2, JAK2, ADAM10, and EMT marker levels in SW620 and HCT116 cells. *p < 0.05; **p < 0.01; ***p < 0.001.

We further sought to examine the signaling pathways whereby miR-365-3p modulated CRC progression. To that end, we assessed the protein levels of p-STAT3, STAT3, p-JAK2, and JAK2 in CRC cells that had been transfected with miR-365-3p mimics and ADAM10 or control expression vectors. These experiments demonstrated that miR-365-3p was able to reduce JAK2 and STAT3 phosphorylation, while ADAM10 overexpression had the opposite effect and reduced the ability of miR-365-3p to suppress the activation of these signaling proteins in CRC cells (**Fig. 6A** and **B**). We further found that miR-365-3p attenuated the expression of proteins associated with the EMT, whereas ADAM10 overexpression was able to counteract this phenotype.

## Discussion

CRC remains one of the most common cancers globally, with 1.3 million new annual cases, and with surgical resection remaining the primary effective mode of treatment 11, 12. Given its prevalence, it is vital that current understanding of the development and progression of CRC be improved 13, 14. Recent research has highlighted the close relationship between altered miRNA expression and the growth, metastasis, and angiogenic development of CRC 15, 16. As such, efforts to better understand the miRNAs which promote or suppress CRC progression may offer invaluable diagnostic and/or therapeutic opportunities.

In the present study, we found that miR-365-3p expression was markedly reduced in both CRC cells and tissue samples, with this downregulation being closely associated with many adverse clinical findings including larger tumor sizes, local invasion, and metastasis to lymph nodes. Those patients expressing lower levels of miR-365-3p also had reduced OS and RFS relative to patients with higher expression of this miRNA. Previous studies have shown miR-365-3p to have prognostic relevance in laryngeal squamous cell carcinoma and hepatocellular carcinoma 17, 18, but it has not previously been examined as a relevant prognostic biomarker in CRC, adding novelty to the present report.

miRNAs are small non-coding RNA molecules that are capable of binding to the 3'-UTR of target mRNAs owing to sequence complementarity, thereby regulating these target genes 19, 20. Herein we assessed the potential targets of miR-365-3p that may be associated with its ability to suppress CRC invasion, proliferation, and metastasis. Through a bioinformatics-based predictive approach, we identified ADAM10 as a putative miR-365-3p target that was of key interest given its previously documented role in cleaving protein substrates at the extracellular membrane 21, 22. Previous work has also shown ADAM10 to be related to neurodegenerative diseases, cancer, and immunological disorders 23, 24. ADAM10 activity is controlled at both the transcription and translational levels 22, 25, 26. A number of distinct transcription factors can bind the ADAM10 promoter to drive its transcription 27, 28. Furthermore, specific miRNAs can suppress ADAM10 expression via binding to the 3'-UTR of ADAM10 mRNA molecules. Altered ADAM10 activity has been detected in many diseases, and has been linked with the pathological processes therein 29, 30. Some researchers have suggested that inhibiting the ability of ADAM10 to mediate protein cleavage may reverse Notch1 signaling in T cell acute lymphoblastic leukemia, thereby suppressing tumorigenesis 29, 31. In HER2+ breast cancer cells, inhibiting or knocking down ADAM10 was similarly shown to enhance Trastuzumab treatment efficacy 32-34. Using qRT-PCR, Western blotting, and luciferase reporter assays, we were able to confirm the ability of miR-365-3p to directly target ADAM10 and to suppress its translation. We further confirmed through rescue experiments that overexpressing ADAM10 was sufficient to reduce the ability of miR-365-3p to suppress CRC metastasis, thus suggesting that miR-365-3p-mediated ADAM10 suppression is at least partially responsible for the ability of this miRNA to regulate CRC development and progression.

Cytokines can, through binding to their cognate cell surface receptors, initiate intracellular signal transduction cascades. JAK-STAT signaling is a key form of signal transduction in response to certain cytokines, mediating the growth and metastasis of cancer cells and other cell types 35_._ In JAK-STAT signaling, cytokines and growth factors mediate receptor dimerization, leading to JAK phosphorylation and subsequent STAT activation. Altered STAT3 activation is known to be a common hallmark of abnormal cellular proliferation and malignancy, leading to substantial interest in such JAK-STAT signaling in the context of oncogenesis 36. Herein we determined that miR-365-3p was able to inhibit JAK-STAT signaling at least in part via targeting ADAM10, thereby suppressing the progression of CRC, thus highlighting a novel mode of JAK-STAT3 regulation.

In summary, we observed reduced levels of miR-365-3p in CRC patient samples and cells. These lower levels were associated with larger tumor sizes, more significant local invasion and metastasis to lymph nodes, and an overall poorer CRC patient prognosis. We further found that miR-365a-3p inhibits JAK/STAT3 signaling and CRC cell metastatic progression via inhibiting ADAM10 expression, suggesting this pathway may be a viable therapeutic target in those with CRC.

## Figures and Tables

**Table 1:** Association between miR-365a-3p level and clinicopathologic characteristics of CRC patients in the study cohort.

Characteristics	No.of patients	miR-365a-3p		p value
		low(N=67)	High (N=67)	
**Gender**				0.862
Female	59	29	30	
Male	75	38	37	
**Age(years**)				0.849
<60	39	19	20	
≥60	95	48	47	
**Tumor location**				0.073
Recturn	60	29	31	
Colon	74	38	36	
**Differentiation grade**				0.685
Well+Moderate	102	50	52	
Poor	32	17	15	
**Tumer size (cm)**				0.022
<5	53	20	33	
≥5	81	47	34	
**Local invasion**				0.043
pT1-T2	102	46	56	
pT3-T4	32	21	11	
**Lymph node metastasis**				0.021
N0+N1	83	35	48	
N2	51	32	19	
**TNM stage**				0.291
Ⅰ+Ⅱ	80	37	43	
Ⅲ	54	30	24	
**CA19-9(kU/L)**				0.604
<37	63	30	33	
≥37	71	37	34	
**Serum CEA level (ng/mL)**				0.166
<5	59	31	28	
≥5	75	36	39	

Carbohydrate Antigen 19-9, CA19-9; Carcinoembryonic Antigen,CEA

**Table 2:** Univariate and multivariate analyses of clinicopathologic parameters associated with recurrence-free survival and overall survival

Variables	Categories	Umivariate analysis				Multivariate analysis			
		HR	95%Cl		p value	HR	95%Cl		p vaule
**Recurrence-Free Survival**									
Gender	Male/female	1.061	0.613	1.158	0.923	-	-	-	NA
Age(years)	≥60/<60	0.811	0.687	0.671	0.345	-	-	-	NA
Tumor location	Colon/recsum	1.241	0.905	1.715	0.143	-	-	-	NA
Tumor size(cm)	≥5/<5	1.365	1.271	1.718	0.025	1.179	1.087	1.397	0.048
Differentiation grade	Poor/well+moderate	1.324	1.198	1.593	0.046	-	-	-	NS
Local invasion	pT3-4/pT1-2	1.831	1.522	1.796	0.014	1.471	1.288	1.655	0.038
Lymph node metastasis	N2/N0+N1	1.792	1.502	2.116	0.019	-	-	-	NS
TNM stage	Ⅲ/Ⅰ+Ⅱ	1.927	1.6901	2.414	0.011	1.569	1.245	2.197	0.019
CA19-9 (KU/L)	≥37/<37	1.066	0.676	1.519	0.156	-	-	-	NA
CEA (ng/mL)	≥5/<5	1.196	1.065	1.468	0.042	-	-	-	NS
miR-365a-3p	low/high	1.361	1.148	1.975	0.022	1.187	1.063	1.641	0.045
**Overall Survival**									
Gender	Male/female	1.162	0.751	1.487	0.939	-	-		NA
Age(years)	≥60/<60	1.138	0.634	1.158	0.432	-	-	-	NA
Tumor location	Colon/recsum	1.097	0.948	1.3671	0.144	-	-	-	NA
Tumor size(cm)	≥5/<5	1.347	1.293	1.715	0.025	1.181	1.066	1.781	0.031
Differentiation grade	Poor/well+moderate	1.154	0.789	1.718	0.035	-	-	-	NS
Local invasion	pT3-4/pT1-2	1.981	1.3835	2.593	0.018	1.207	1.186	1.718	0.027
Lymph node metastasis	N2/N0+N1	2.463	1.892	2.796	0.002	1.434	1.389	1.759	0.014
TNM stage	Ⅲ/Ⅰ+Ⅱ	2.515	1.782	2.816	0.001	1.132	1.089	1.878	0.035
CA19-9 (KU/L)	≥37/<37	1.514	0.782	1.941	0.064	-	-	-	NA
CEA (ng/mL)	≥5/<5	1.178	1.082	1.519	0.043	-	-	-	NS
miR-365a-3p	low/high	1.473	1.334	1.683	0.024	1.277	1.128	1.661	0.039

Note. NA: not adopted; NS: not significant.
