# miR-365a-3p regulates ADAM10-JAK-STAT signaling to suppress the growth and metastasis of colorectal cancer cells: Erratum

**DOI:** 10.7150/jca.79828

**Published:** 2023-01-12

**Authors:** Yong-gang Hong, Cheng Xin, Ji-dian Zhou, Xian-hua Gao, Lun Hao, Qi-zhi Liu, Wei Zhang, Li-qiang Hao

**Affiliations:** 1 Department of Colorectal Surgery, Changhai Hospital, Second Military Medical University Shanghai, P.R. China, 200433; 2 Pella Christian High School, Iowa, United States of America.

In the initially published version of our article, we recently realized that there are errors in several figures and Table 2 due to misplace of images and data. The corrections are provided below.

Finally, the author list in the paper should be updated as above in this erratum.

The correction does not change the overall conclusions of this paper. We apologize for the error and for any inconvenience that may cause to the readers and the editors of this journal.

## Figures and Tables

**Figure 1:**
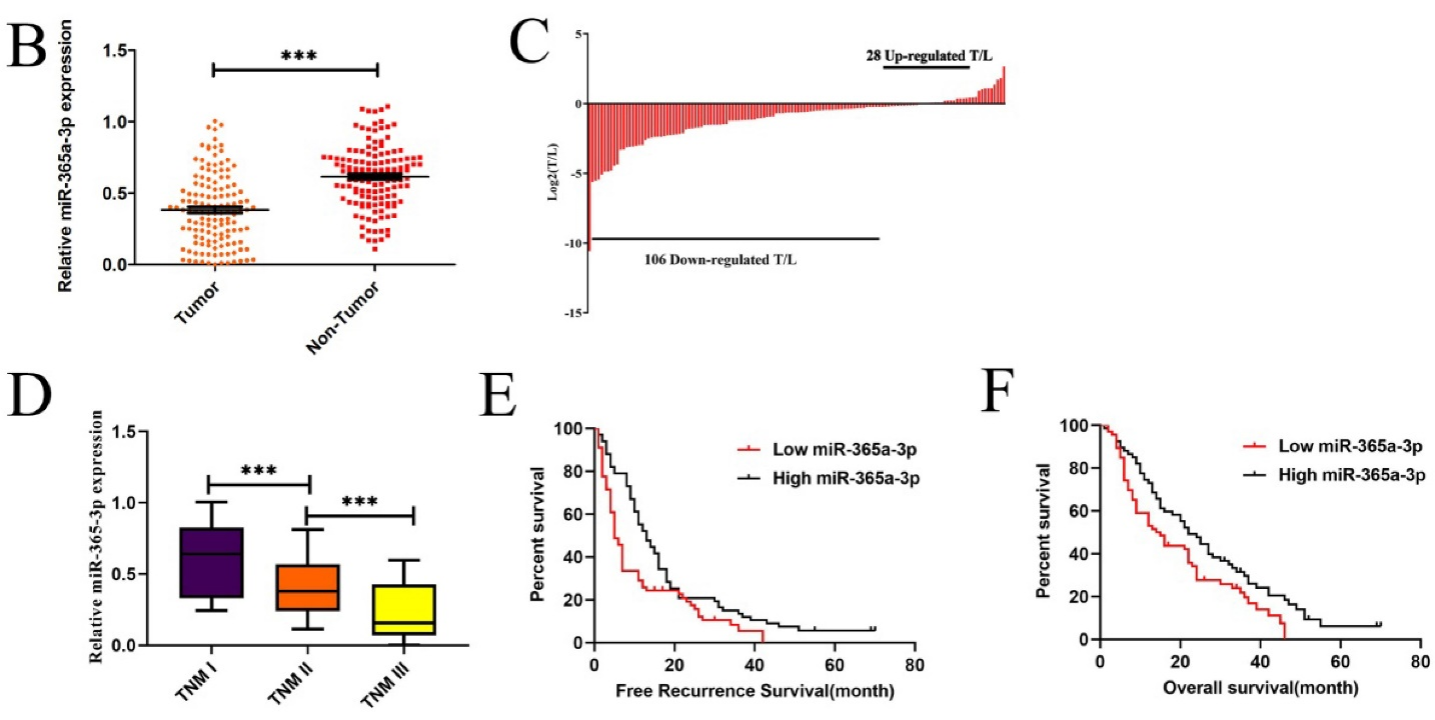
** Corrected Figure 1B-F.** (**B**-**C**) Relative miR-365a-3p levels in 134 pairs of primary CRC patient tumor and paracancerous tissue as accessed via qRT-PCR. (T, tumor; ANT, adjacent nontumor tissue). (**D**) A gradual reduction in levels of miR-365a-3p was evident with increasing TNM stage (I-III). (**E and F**) The association between levels of miR-365a-3p and recurrence-free survival (RFS,** E**) and overall survival (OS,** F**) in these 134 patients was measured via the Kaplan-Meier method. *p < 0.05; ***p < 0.001.

**Figure 3:**
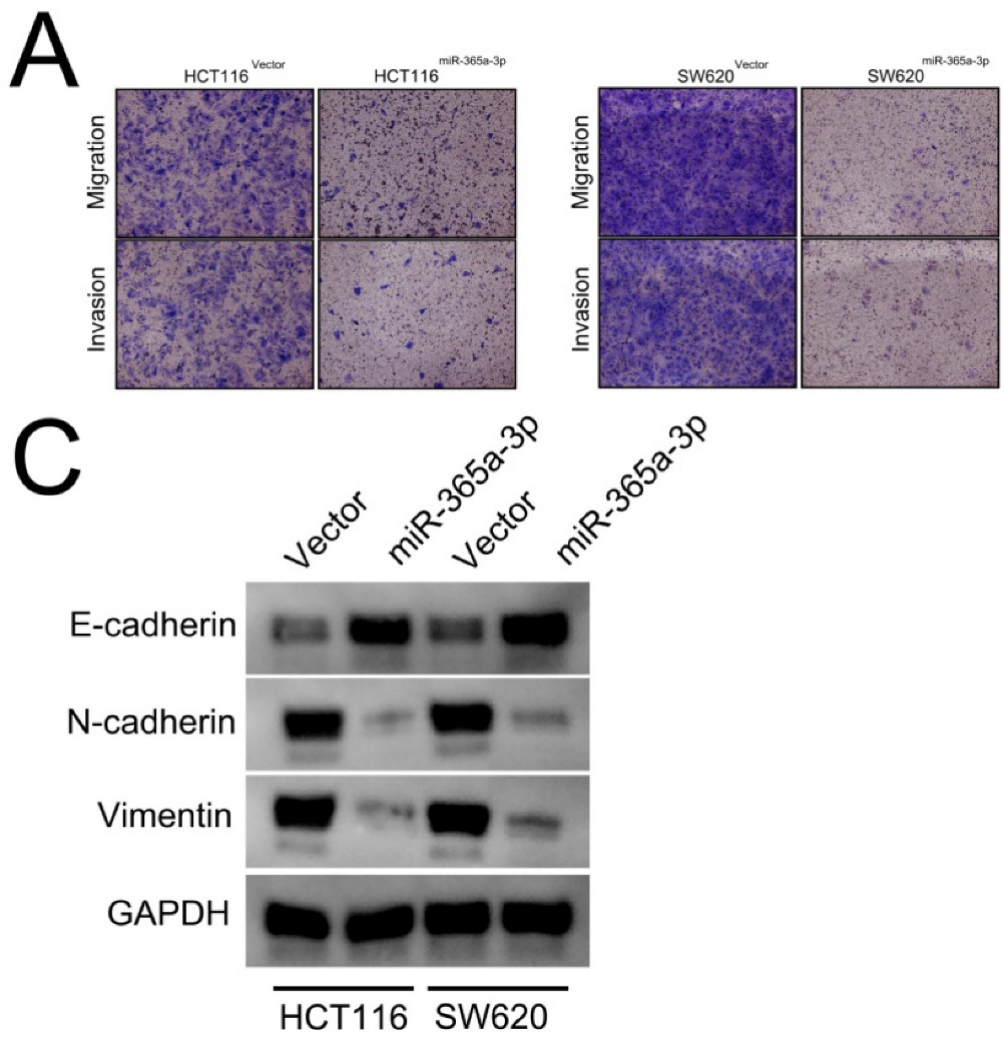
** Corrected Figure** **3A and C.** (**A**) Transwell-based invasion and migration assays were used to assess the impact of miR-365-3p overexpression on HCT116 and SW620 cell migratory and invasive activity, with representative images shown. (**C**) Levels of E-cadherin, N-cadherin, and vimentin were measured via Western blotting in CRC cells overexpressing miR-365a-3p.

**Figure 5:**
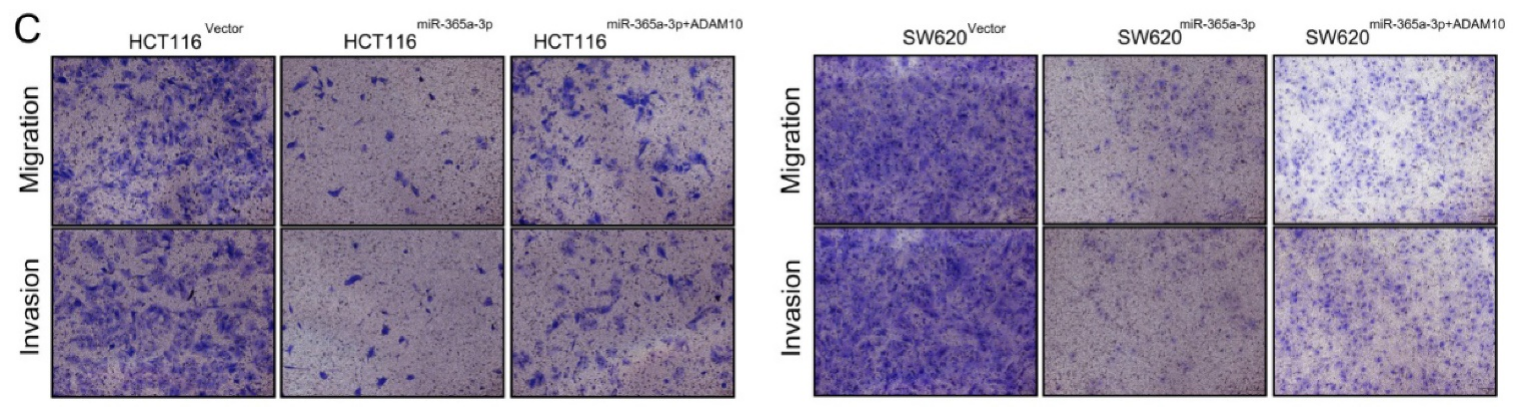
** Corrected Figure 5C.** (**C**) HCT116 cell migration was assessed via Transwell assay following the indicated treatment combinations.

**Figure 6:**
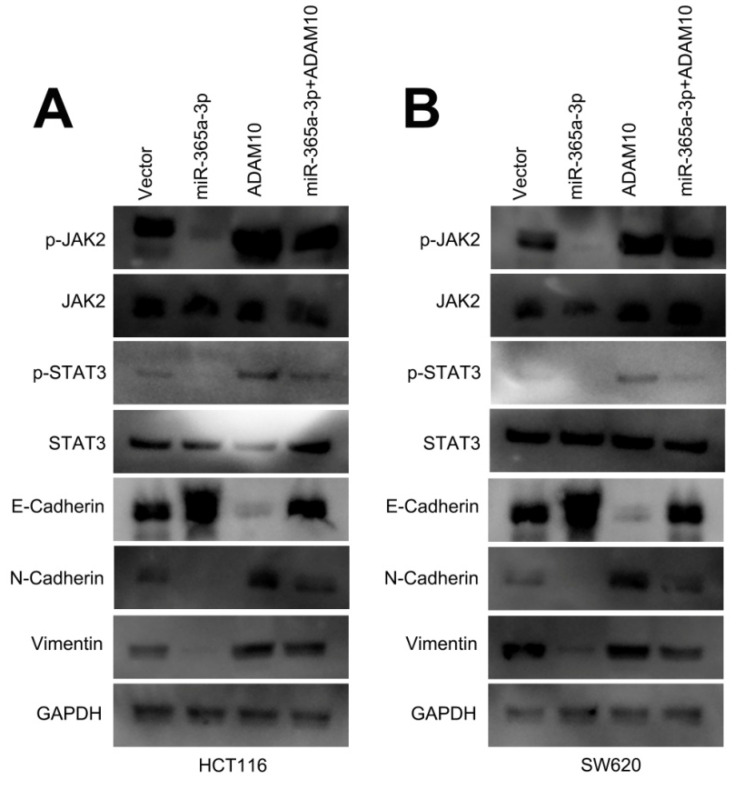
** Corrected Figure** **6. miR-365-3p regulates JAK/STAT signaling to inhibit CRC progression.** (**A** and **B**) Western blotting as used to assess p-STAT3, STAT3, p-JAK2, JAK2, and EMT marker levels in SW620 and HCT116 cells.

**Table 2:**
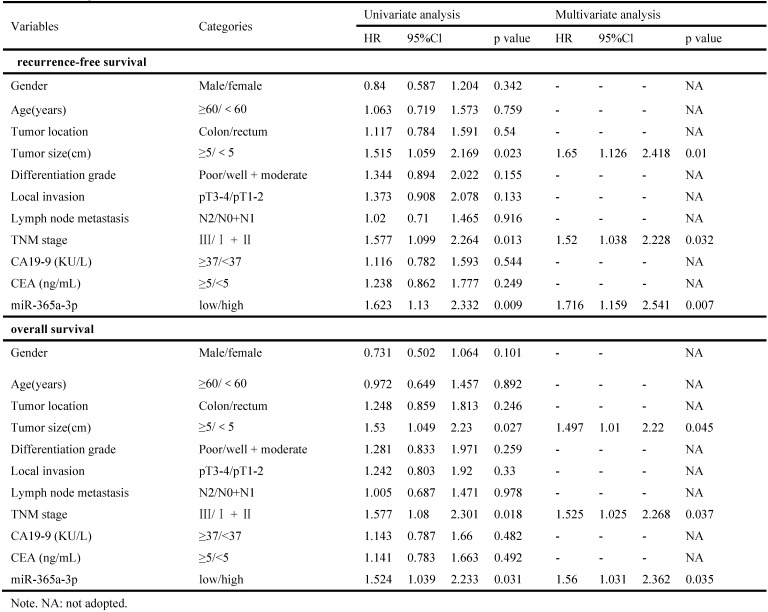
Corrected Table 2. Univariate and multivariate analyses of clinicopathologic parameters associated with recurrence-free survival and overall survival

